# A novel ulvan lyase family with broad-spectrum activity from the ulvan utilisation loci of *Formosa agariphila* KMM 3901

**DOI:** 10.1038/s41598-018-32922-0

**Published:** 2018-10-02

**Authors:** Venkat Rao Konasani, Chunsheng Jin, Niclas G. Karlsson, Eva Albers

**Affiliations:** 1 Industrial Biotechnology Division, Department of Biology and Biological Engineering, Chalmers University of Technology, Gothenburg, Sweden; 2 Department of Medical Biochemistry and Cell Biology, Institute of Biomedicine, University of Gothenburg, Gothenburg, Sweden

## Abstract

Ulvan, which is one of the major structural polysaccharides of the cell walls of green macroalgae, is degraded by ulvan lyases *via* the β-elimination mechanism with the release of oligosaccharides that have unsaturated 4-deoxy-L-threo-hex-4-enopyranosiduronic acid (∆) at the non-reducing end. These ulvan lyases belong to the PL24 or PL25 or PL28 family in the CAZy database. In this study, we identify and biochemically characterise a periplasmic novel broad-spectrum ulvan lyase from *Formosa agariphila* KMM 3901. The lyase was overexpressed in *Escherichia coli*, and the purified recombinant enzyme depolymerised ulvan in an endolytic manner with a *K*_*m*_ of 0.77 mg/ml, and displayed optimum activity at 40 °C and pH 8. This lyase also degraded heparan sulphate and chondroitin sulphate. Detailed analyses of the end-products of the enzymatic degradation of ulvan using ^1^H- and ^13^C-NMR and LC-MS revealed an unsaturated disaccharide (∆Rha3S) and a tetrasaccharide (∆Rha3S-Xyl-Rha) as the principal end-products. In contrast to the previously described ulvan lyases, this novel lyase is mostly composed of α-helices that form an (α/α)_6_ incomplete toroid domain and displays a remarkably broad-spectrum activity. This novel lyase is the first member of a new family of ulvan lyases.

## Introduction

About a half of the photosynthesis that takes place on Earth occurs in aquatic plants, with green macroalgae being significant contributors to aquatic photosynthesis. Green macroalgae, especially members of *Ulvales*, are infamously known for their growth in eutrophicated waters, creating “green tides,” which are an environmental problem and an economic burden for coastal municipalities^1^. Currently, *Ulva lactuca* biomass, which is rich in protein, minerals, and dietary fibre, is consumed as “sea lettuce.” However, this consumption represents only a minor fraction of available green macroalgae biomass. Moreover, since green macroalgae biomass is rich in polysaccharides, it has potential as an alternative renewable energy source if used as a feedstock in biofuel production, e.g., in acetone-butanol-ethanol fermentation^2,3^. A major obstacle to the valorisation of this biomass is the lack of specific tools for saccharification of its polysaccharides. The enzymes that have been developed for the saccharification of polysaccharides of terrestrial plants are not efficient at saccharifying algal polysaccharides due to the complexity of the latter. The high salinity, readily available anions such as sulphate and phosphate in marine water and the adaptations of the algae to these conditions result in the covalent modifications of the polysaccharides that contribute to the complexity. Moreover, marine polysaccharides are often composed of rare sugars, e.g. iduronic acid in ulvan. Therefore, there is a need to develop specific enzymatic tools for the depolymerisation of green seaweed polysaccharides.

**Figure 1:**
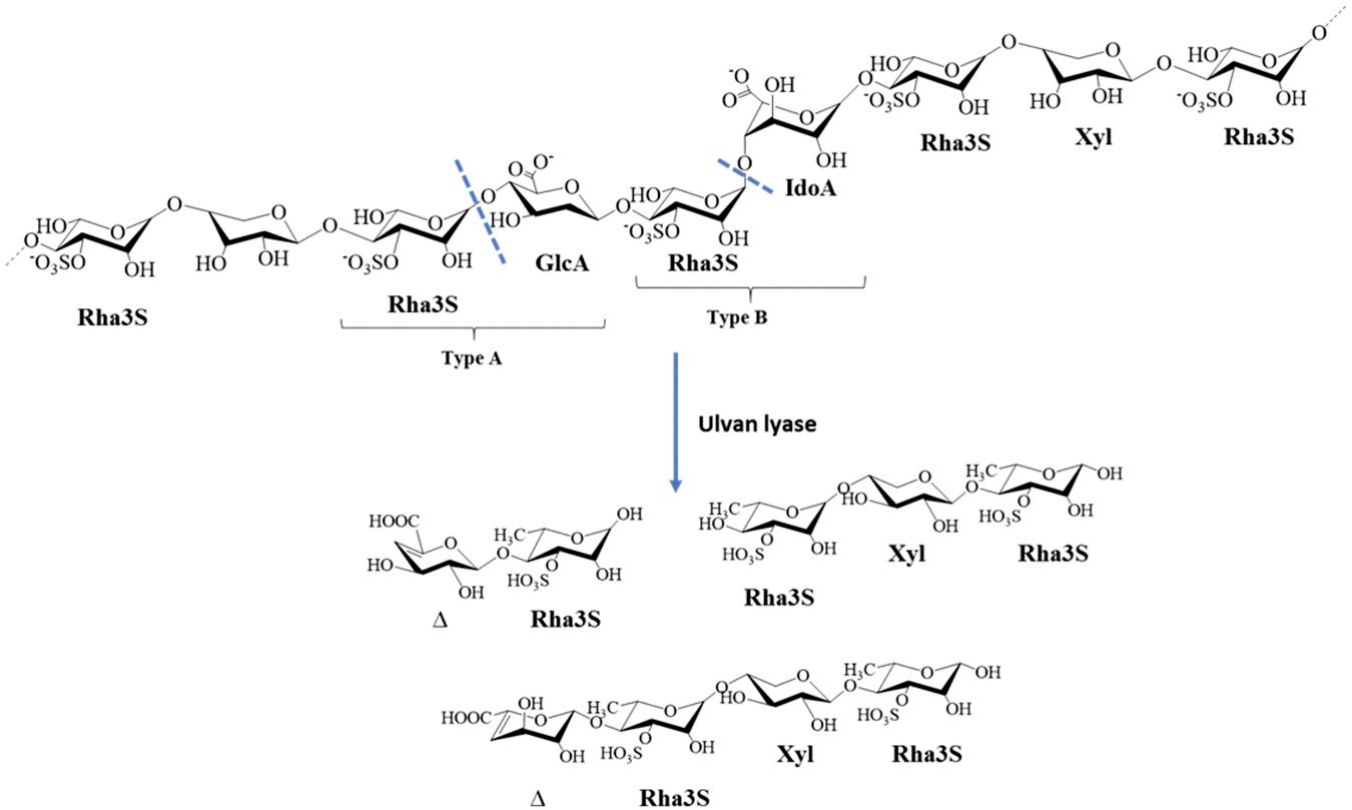
The structure of ulvan and the mode of action of ulvan lyase. The dotted blue lines indicate the points of action of the ulvan lyase.

Members of the green macroalgae family synthesise several polysaccharides. Among these, ulvan is a major structural polysaccharide in the cell wall, constituting up to 29% of the dry weight of the alga^4^. The major constituents of ulvan are rhamnose-3-sulphate (Rha3S), glucuronic acid (GlcA), iduronic acid (IdoA), and xylose (Xyl). The structure of ulvan is complex, comprising two primary repeating disaccharide moieties: ulvanobiuronic acid-3-sulphate Type A [β-D-GlcA (1,4) α-L-Rha3S]; and Type B [α-L-IdoA (1,4) α-L-Rha3S]^5^. Another minor repeating disaccharide moiety contains Rha3S linked to Xyl, which replaces the uronic acid (Fig. 1). Ulvan displays several physicochemical and biological properties with potential applications as food/feed ingredients, pharmaceuticals, and biomaterials, as well as plant immunomodulators and growth promoters^6–10^. Ulvan is also a source of the rare sugar iduronic acid, which has applications in the chemical synthesis of heparin analogs^11^.

Enzymatic depolymerisation of polysaccharides takes place primarily *via* hydrolysis and lytic β-elimination^12^. Lytic β-elimination occurs in polysaccharides that contain uronic acid, e.g., ulvan, and involves polysaccharide lyases (PLs). PLs are classified into 28 PL families in the Carbohydrate-Active enZYmes database (CAZy) based on sequence (www.cazy.org)^13^. To date, only a few ulvan lyases have been reported, and some of these have been distributed into three families: PL24, PL25, and PL28 family. The first ulvan lyase was reported by Lahaye *et al*. in a marine Gram-negative bacterium isolated from mud that contained decomposing *Ulva*^14^. Apart from its use in the structural characterisation of ulvan, no further details of its properties were provided. Subsequently, a marine bacterium belonging to the phylum Bacteroidetes, *Nonlabens ulvanivorans*, was reported to have the ability to degrade ulvan^15^. The ulvan lyase from this bacterium was purified and characterised by Collen *et al*. and was reported to cleave the glycosidic bond between Rha3S and GlcA or IdoA^16^. Recently, other ulvan lyases without homology to the *N*. *ulvanivorans* ulvan lyase have been reported for members of the *Alteromonadales* order^17^. These lyases were overexpressed in *Escherichia coli* and biochemically characterised. In contrast to the *N*. *ulvanivorans* ulvan lyase, the ulvan lyase of *Alteromonadales* is highly specific for the glycosidic bond between Rha3S and GlcA. Most recently, Ulaganathan *et al*. unravelled the crystal structures of three ulvan lyases, one representative from each PL2, PL25 and PL28 family, and postulated the convergent evolution of the ulvan lyase active site^18–20^. Despite their low-level homology, the structures of the PL24 and PL25 family ulvan lyases share a similar seven-bladed β-propeller conformation. PL28 family ulvan lyase displays a distinct β-jelly roll fold.

The polysaccharide depolymerisation system comprises specific carbohydrate-active enzymes, and sensing and transporting proteins. In Gram-negative bacteria, specifically Bacteroidetes, the genes for these proteins are organised into polysaccharide utilisation loci (PUL)^21^. To date, two PUL responsible for ulvan depolymerisation have been reported: one from *Alteromonas* sp. and the other from *Formosa agariphila*^22,23^. The *Alteromonas* ulvan PUL expresses two different ulvan lyases, assigned to the PL24 and PL25 families. In contrast, only one ulvan lyase has been identified in *F*. *agariphila*, which is a marine algae-associated heterotrophic bacterium that has the potential to utilise various algal polysaccharides^24^. Salinas and French (2017) recently showed that this bacterium could utilise ulvan, and they identified an ulvan utilisation loci (PUL for ulvan) that encode an ulvan lyase (GenBank accession no. Cdf79931)^23^. As mentioned earlier, ulvan is a complex polysaccharide, so the ulvan utilisation loci express several proteins. However, functional properties of many of these proteins are still not known. In this study, we identified a novel broad-spectrum ulvan lyase (GenBank accession no. Cdf79930) from this PUL for ulvan of *F*. *agariphila* and examined its role in ulvan depolymerisation.

## Results

### *In silico* analyses

#### Sequence analysis

**Figure 2:**
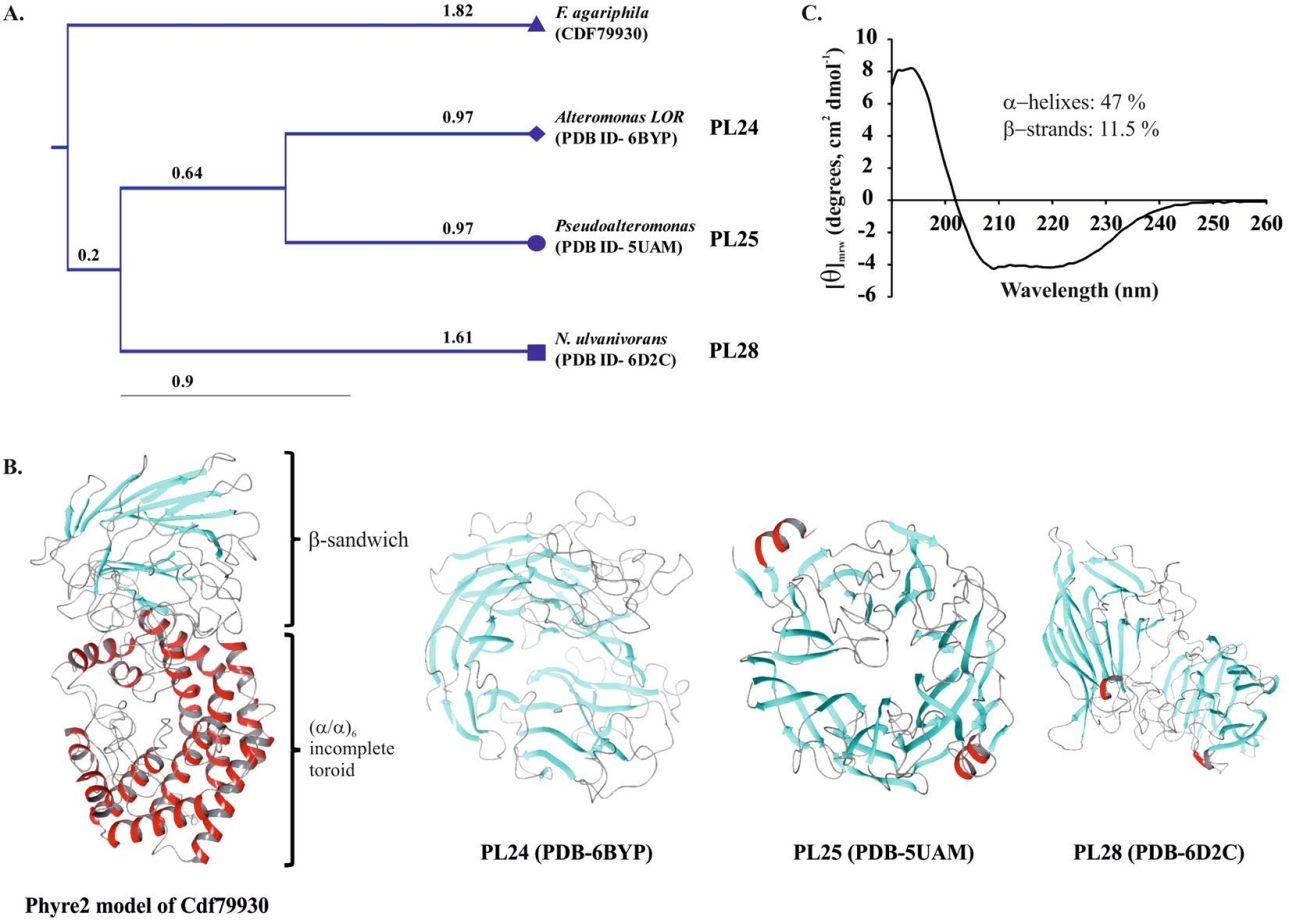
*In silico* analyses of Cdf79930. (**A**) Phylogenetic analysis of ulvan lyases. This phylogenetic tree was constructed using UPGMA based on a Kimura 2p distance matrix using the program CLC Sequence Viewer ver. 8.0. Numbers indicate the bootstrap values. (**B**) Structures of ulvan lyases. (**C**) The CD spectrum of Cdf79930. The CD measurements were carried out using 0.1 mg ml^−1^ solution of Cdf79930 in 10 mM Tris buffer (pH 8). The average of the three scans was plotted, and the secondary structure (inset) was calculated using the K2D2 server.

The Cdf79930 protein does not share homology with the previously characterised ulvan lyases; instead, forms an evolutionarily distinct new family (Fig. 2A). Moreover, PSI-BLAST (5 iterations) searching for this protein sequence generated similar putative proteins that are present in several bacterial phyla, particularly the Bacteroidetes phylum, which represented 78% of the sequence hits. Among the PSI-BLAST hits, proteins up to 30% identity to Cdf79930 were all hypothetical, and none of them was previously characterised. PSI-BLAST also revealed the presence of many homologs of Cdf79930 in the two major phyla found in the human gut microbiota, i.e., Bacteroides and Firmicutes (Fig. S1). Surprisingly, this is the first report on the presence of homologs of ulvan lyase in the human gut microbiota, as the PSI-BLAST search for homologs of the previously identified ulvan lyases did not reveal any homologs in the human gut microbiota. In Bacteroides, interestingly, the homolog of Cdf79930 is located in the PUL for heparin, whereas in firmicutes it lies in proximity to the genes that encode chondroitin catabolizing enzymes.

#### Protein structure analysis

The secondary structure prediction with PSI-PRED indicated two distinct regions in Cdf79930: an α-helix-rich N-terminal region (also predicted by InterPro); and a β-strand-rich C-terminal region. Similarly, secondary structure analysis of Cdf79930 by CD spectroscopy revealed 42% α-helices and 11.52% β-strands (Fig. 2C). Using Phyre2, more than 92% of the residues were modelled at >90% confidence, and the resulting 3D model further revealed the distinct conformation, i.e., an (α/α)_6_ incomplete toroid at the N-terminus and a C-terminal β-sandwich domain (Fig. 2B).

### Heterologous expression, purification, and biochemical characterisation

**Figure 3:**
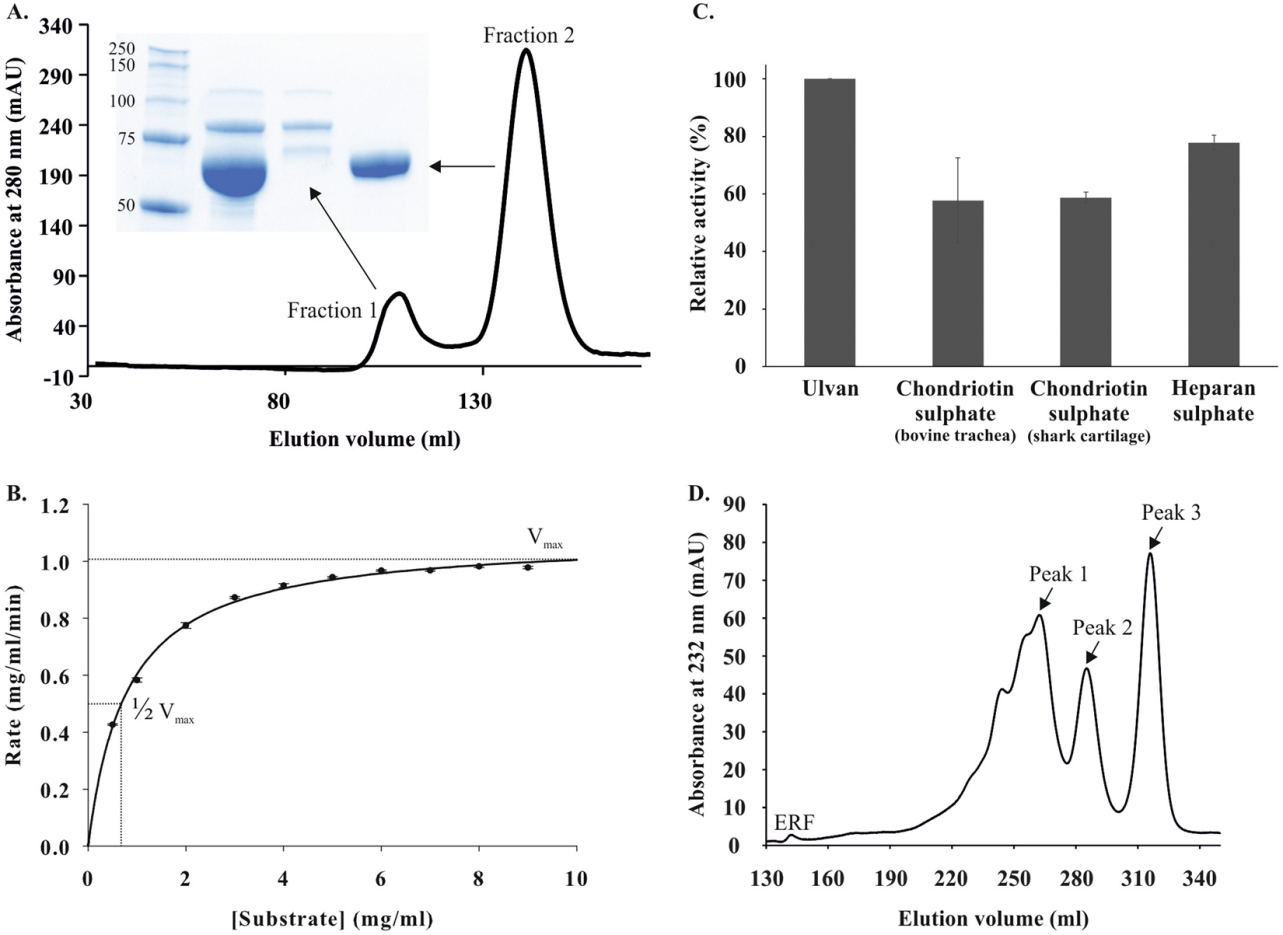
Purification of the Cdf79930 and its activity against ulvan. (**A**) Purification of Cdf79930 by size exclusion chromatography and SDS-PAGE analysis of the purified fractions: lane 1, molecular weight standard; lane 2, Cdf79930 fraction after immobilised metal affinity chromatography; lane 3, fraction 1 after size exclusion chromatography; and lane 4, fraction 2 after size exclusion chromatography. The picture of the SDS-PAGE gel was cropped; full gel picture is added as Fig. S7 in the Supplementary Information. (**B**) Michaelis-Menten plot of Cdf79930 with ulvan extracted from *U*. *amoricana* as substrate. Each point represents the average value of triplicate measurements, and the error bar indicate the standard error. The fitted *K*_*m*_ and *V*_*max*_ values are 0.77 mg ml^−1^ and 540 µg of reducing end equivalents of glucose ml^−1^ min^−1^ mg protein^−1^, respectively. (**C**) Substrate specificity of the novel ulvan lyase. Cdf79930 was incubated with 200 µg of each substrate and incubated for 60 minutes at 37 °C. Cdf79930 depolymerised both chondroitin sulphate and heparan sulphate. (**D**) Separation of end-products from the Cdf79930 action on ulvan using size exclusion chromatography.

Sequence analysis using the SignalP and PrediSi web software tools indicated the presence of a 15-amino acid signal peptide at the N-terminus, and the protein as localised in periplasm^25,26^. The Cdf79930 protein was expressed without this signal peptide, and the expression of soluble Cdf79930 was obtained at 16 °C. The recombinant protein was purified first using an immobilised metal (Ni^2+^) affinity column and thereafter passed through a size exclusion chromatography column. In the size exclusion chromatography, ulvan lyase activity was eluted as a single peak, and the eluted protein was homogeneous, as observed using denaturing polyacrylamide gel electrophoresis (Fig. 3A).

**Figure 4:**
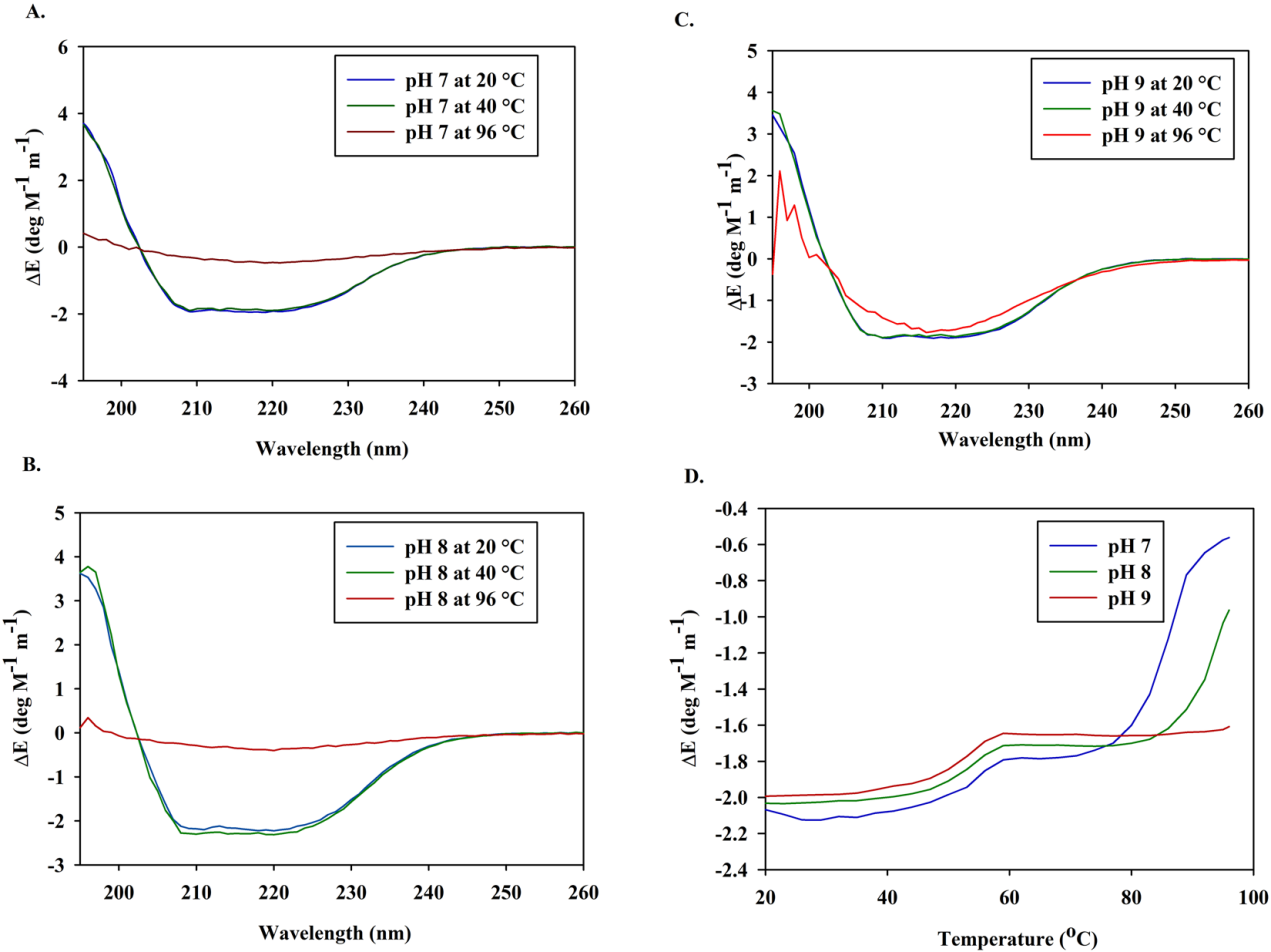
CD measurements of Cdf79930. CD spectra of Cdf79930 measured in 10 mM tris buffer pH 7 (**A**), pH 8 (**B**), pH 9 (**C**), and at different temperatures 20 °C, 40 °C, 96 °C. Thermal denaturation (**D**) was carried out at 222 nm in 10 mM tris buffer pH 7, 8 and 9.

Biochemical characterisations were performed on the purified samples of Cdf79930 obtained after size exclusion chromatography (Table 1). Cdf79930 was optimally active at 40 °C. The addition of up to 500 mM sodium chloride did not have a significant effect on the ulvan lyase activity (Table 1). The *V*_*max*_ and *K*_*m*_ values of ulvan lyse were 540 µg of reducing ends/ml. min. mg protein and 0.77 mg/ml, respectively (Fig. 3B). Cdf79930 degraded both chondroitin sulphate and heparan sulphate (Fig. 3C). However, no detectable lyase activity was observed against pectin, xanthan or alginate. The initial velocity as a function of the pH curve was bell-shaped, and the maximum initial velocity was observed at pH 8.0. A loss of 50% of activity was observed at pH 7.0 and pH 9.0. The conformation of the enzyme changed from pH 7.0 to 8.0, while it displayed similar conformation at pH 8.0 and 9.0 (Fig. 4A–C). Consistent with this, pH also affected the thermal denaturation of the Cdf79930 (Fig. 4D). The enzyme displayed a biphasic thermal denaturation due to the presence of two distinct domains and showed the higher melting temperature at the alkaline pH.

**Table 1:** Biochemical properties of Cdf79930^a^.

*K*_*m*_	0.77 mg ml^−1^
*V*_*max*_	540 µg of glucose ml/min/mg protein
Optimum temperature	40 °C
Optimum pH	8.0
Effect of NaCl (0–500 mM)	No effect

All the measurements were carried out in triplicate, and the average value is presented. Ulvan from *U*. *amoricana* was used for all the biochemical characterisation experiments.

### Purification and analyses of the end-products

**Figure 5:**
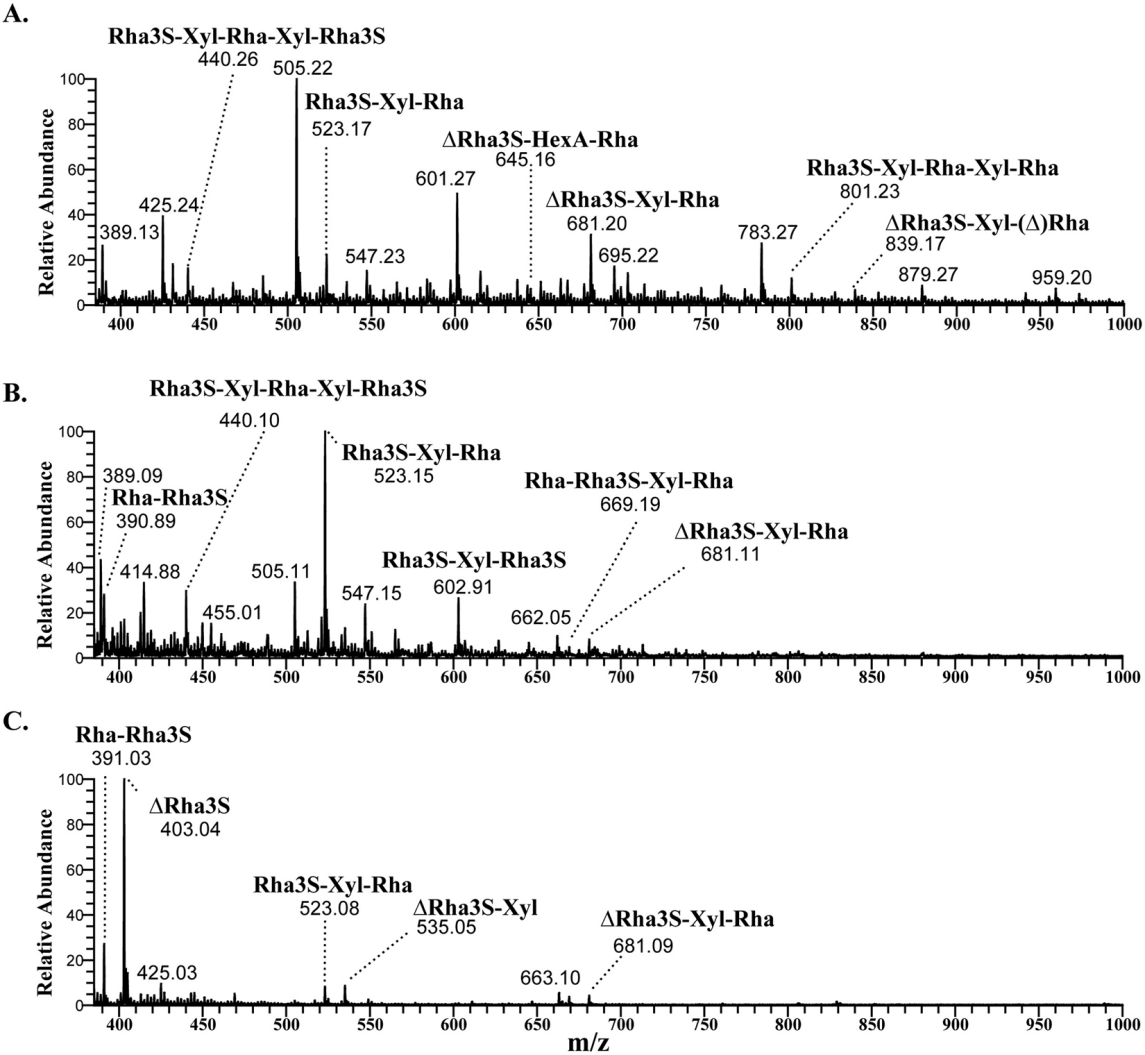
Mass spectrometry analyses of the oligosaccharide end-products of Cdf79930-digested ulvan, separated using size exclusion chromatography. Oligosaccharide composition of the peaks collected during size exclusion chromatography is identified: Peak 1 (**A**); Peak 2 (**B**); and Peak 3 (**C**).

The recombinant Cdf79930 acted on ulvan and released oligosaccharides. The time course of ulvan depolymerisation was monitored using absorption at 232 nm, which is typical for -C=C-unsaturation, on a size exclusion chromatography column. Cdf79930 rapidly attacked the ulvan in an endolytic fashion and initially generated long chains of ulvan with unsaturated non-reducing ends. The sizes of the released oligosaccharides decreased with increases in the incubation time. After 12 hours of incubation with a two-fold concentration of enzyme, the ulvan was completely depolymerised to small oligosaccharides (Fig. S2). These oligosaccharide end-products were separated by size exclusion chromatography into three distinct peaks (Fig. 3D). The oligosaccharides in these peaks were analysed by ^1^H- and ^13^C-NMR and mass spectrometry. The mass spectrometry analyses revealed that the peaks separated by size exclusion chromatography were not pure, but were instead a mixture of oligosaccharides (Fig. 5). Peak 1 (K_av_ 0.29) predominantly consisted of a mixture of tetrasaccharides, ∆Rha3S-Xyl-Rha, ∆Rha3S-(GlcA/IdoA)-Rha, and the pentasaccharides, Rha3S-Xyl-Rha-Xyl-Rha(3S), ∆Rha3S-Xyl-Rha-∆ (Fig. 5A). Further fragmentation analyses of the pentasaccharide peaks on MS/MS indicated branching on the terminal Rha(3S) (Figs S3, S4). The branching residue was deemed to be either saturated or unsaturated hexuronic acid. Peak 2 (K_av_ 0.34) contained mainly the trisaccharide Rha3S-Xyl-Rha and minor portions of ∆Rha3S-Xyl-Rha (Fig. 5B). Peak 3 (K_av_ 0.4) contained mainly a disaccharide (approximately 90%) plus a minor proportion of a trisaccharide. Further analysis of these di- and trisaccharides through fragmentation in LC-MS/MS confirmed that they were: an unsaturated hexopyranosiduronic acid linked to Rha3S (∆Rha3S); and a trisaccharide with Rha(3S) flanking a xylose (Rha3S-Xyl-Rha) (Figs 5C and S3A,B). The separated oligosaccharide end-products were incubated further with the excess Cdf79930. However, no further degradation of these oligosaccharides was observed.

**Figure 6:**
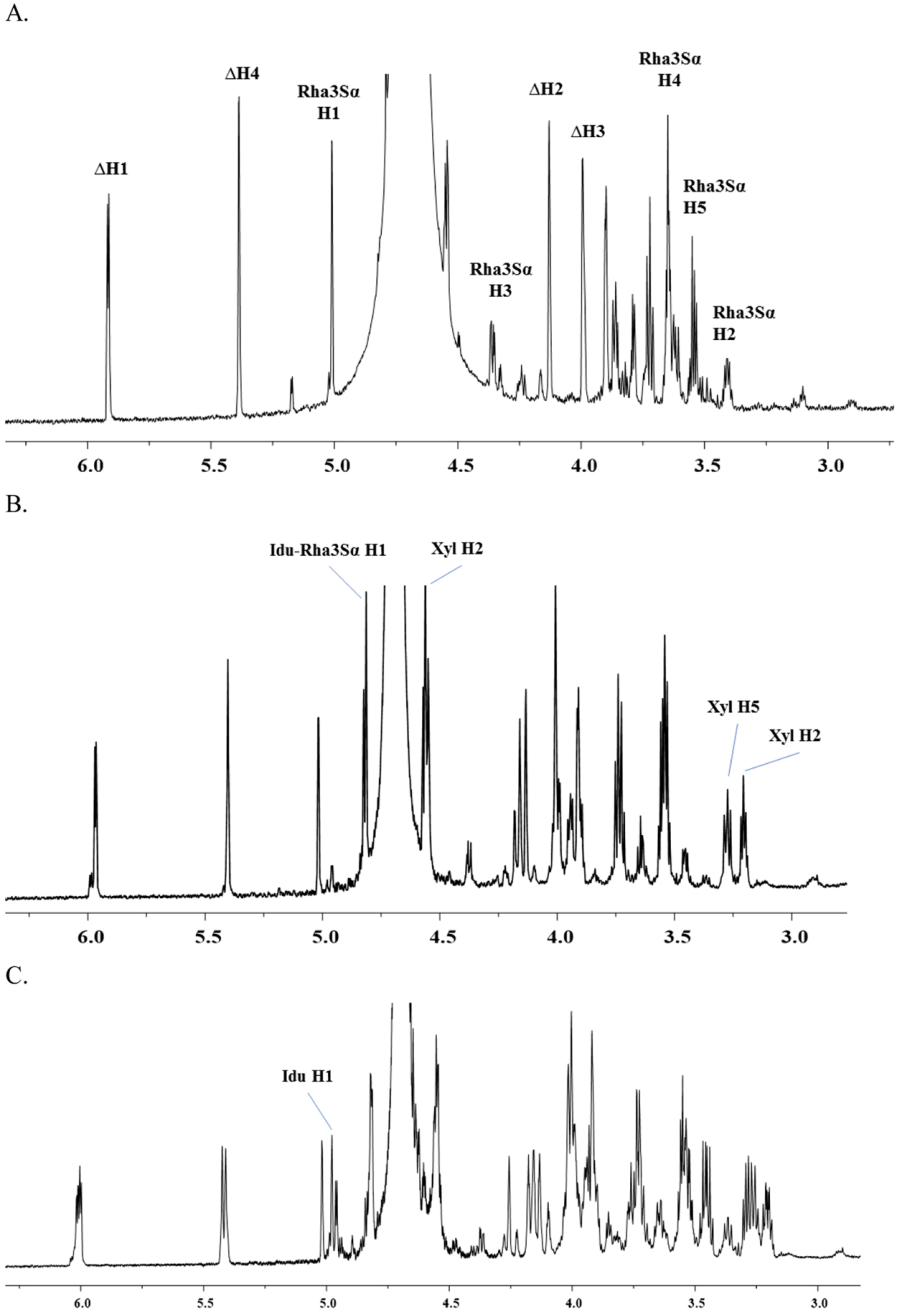
^1^H-NMR spectra of the oligosaccharide end-products of Cdf79930-treated ulvan, separated by size exclusion chromatography. Shown are the ^1^H-NMR spectra for peak 3 eluted between 305 and 328 ml (**A**); peak 2 eluted between 277 and 300 ml (**B**); and peak 1 eluted between 258 and 274 ml (**C**).

The ^1^H-NMR spectrum of peak 3 was compared to the spectrum reported by Lahaye *et al*.^14,27^, and the peak shifts were assigned (Fig. 6). Two prominent signals at 5.97 ppm (∆H1) and 5.4 ppm (∆H4), which are characteristic of ulvan lyase activity, appeared in all the oligosaccharide fractions collected from the size exclusion chromatography, indicating that the oligosaccharides have an unsaturated bond at the non-reducing end. 2D ^1^H- and ^13^C-NMR spectra of the separated peaks were also acquired (Fig. S5).

## Discussion

*Formosa agariphila* genome revealed the potential of this bacterium to utilise several algal polysaccharides, and the presence of a putative ulvan lyase gene- BN863_22190^23,24^. Salinas and French (2017) further confirmed the product of this putative gene as an extracellular ulvan lyase and the presence of a PUL for ulvan. Due to the complexity of ulvan, its depolymerisation needs several enzymes that act at different degrees of polymerisation and at different subcellular locations. Among the 41 proteins encoded by this PUL, except the extracellular ulvan lyase, the majority of the proteins were predicted to be localised to the outer membrane or periplasm. This indicates that most of the steps in the catabolism of ulvan take place at these subcellular locations. In this study, we identified a periplasmic ulvan lyase (encoded by the gene locus- BN863_22180) that plays a crucial role in further depolymerisation of the ulvan oligosaccharides trapped in the periplasm after the initial depolymerisation by the extracellular ulvan lyase and subsequent transport into the periplasm. The gene locus (BN863_22180) was cloned and heterologously expressed in *E*. *coli*. The resulting protein (Cdf79930) was found to be a novel broad-acting ulvan lyase without homology to the previously reported ulvan lyases. The PSI-BLAST search of Cdf79930 and analysis of the PUL for ulvan indicated that homologs of Cdf79930 are present in many bacteria. However, surprisingly, no Cdf79930 homolog was found among the members of the *Alteromonadales* order, even though they are known producers of ulvan lyases. This suggests that there are two different types of PUL for ulvan: (i) *Alteromonadales-*like PUL for ulvan (Fig. S6A); and (ii) *Formosa-*like PUL for ulvan (Fig. S6B). Cdf79930 exhibits an α-helix-rich secondary structure, whereas the ulvan lyases of the *Alteromonadales* order are mostly composed of β-sheets. A homology model of the Cdf79930 protein revealed an (α/α)_6_ incomplete toroid at the N-terminus and a small β-sandwich domain at the C-terminus. In agreement with the homology model, two transitions in the thermal denaturation of Cdf79930 clearly indicate the presence of two different domains. As proposed by Batey and coworkers (2008), and Gupta and coworkers (2014), the melting pattern of Cdf79930 indicates that the unfolding of each domain is independent of the other domain^28,29^. It is most likely that the (α/α)_6_ incomplete toroid domain melts prior to the C-terminal β-sandwich domain. A similar (α/α)_6_ toroid and the β-sandwich domain has been observed for the members of the PL8 family (chondroitin lyase AC, chondroitin lyase ABC, and xanthan lyase), PL15 family (oligo-alginate lyase), and PL21 family (heparinase II)^30,31^. To date, only three structures of ulvan lyases have been reported: one representative from each PL24 (LOR_7 from *Alteromonas*), PL25 (PLSV_3936 from *Pseudoalteromonas*) and PL28 family (NLR48 from *Nonlabens*)^18–20^. Contrasting to Cdf79930, all these ulvan lyases have a structure with the β-fold majority. The PL24 and PL25 family ulvan lyases have a seven-bladed β-propeller conformation while PL28 has a distinct -jelly roll conformation. Despite significant differences in their structures, the spatial arrangements of the catalytic residues of the PL24 and the PL25 family ulvan lyase are similar indicating the convergent evolution of these two families.

The Michaelis-Menten constant for Cdf79930 was calculated as 0.77 mg/ml (Table 1), which is higher than the *K*_*m*_ of the *N*. *ulvanivorans* ulvan lyase, reported as 0.005 mg/ml of ulvan from *U*. *rotundata*^15^. The *N*. *ulvanivorans* ulvan lyase contains a carbohydrate binding module as reported by Melcher *et al*.^32^. However, Cdf79930 is a novel ulvan lyase and has no carbohydrate biding module as analysed using the InterPro, dbCAN (from CAZy database) online analysis tools. This lack of the carbohydrate binding module could be a reason for the higher apparent *K*_*m*_ of the Cdf79930. The *K*_*m*_ values of the ulvan lyases isolated from *Pseudoalteromonas* (2.1 mg/ml), *Glaceicola* (4.1 mg/ml of ulvan from *U*. *ohnoi*), and *Alteromonas* (7.2 mg/ml of ulvan from *U*. *ohnoi*) are much higher than that of the novel ulvan lyase^33–35^. These differences in kinetic properties could be due to the differences in their structure and the differences in the composition of the substrates used. The higher *K*_*m*_ values of the ulvan lyases from *Glaceicola* and *Alteromonas* could be due to their specificity for the β-(1,4) glycosidic bond in Rha3S-GlcA.

Cdf79930 acted in an endolytic fashion and initially formed high-molecular-weight oligosaccharides. The sizes of these oligosaccharides rapidly decreased as the incubation progressed, and small oligosaccharides were formed, with a disaccharide and a tetrasaccharide as the principal end-products. Xylose-containing oligosaccharides, i.e., a trisaccharide and a pentasaccharide, were also formed, although they were not very abundant. The low abundance of these oligosaccharide end-products reflects the monosaccharide composition of the parent ulvan polysaccharide used. The level of xylose is usually lower than those of rhamnose and uronic acids in the ulvan isolated from *U*. *lactuca*. The mass spectrometry and ^1^H-NMR analyses indicated that the end-products were ∆Rha3S, Rha3S-Xyl-Rha, ∆Rha3S-Xyl-Rha, and ∆Rha3S-Xyl-Rha-∆. In similarity to the ulvan lyase of *N*. *ulvanivorans*, reducing ends of the principal end-products of Cdf79930 terminated with Rha(3S), and non-reducing ends with 4-deoxy-L-threo-hex-4-enopyranosiduronic acid which is formed irrespective of the uronic acid partner in the parent polysaccharide^16^. However, the end products of the ulvan lyase of *N*. *ulvanivorans* had two additional tetrasaccharides ∆Rha3S-GlcA-Rha3S and ∆Rha3S-IdoA-Rha3S which were absent in the Cdf79930 end products. The identities of the unsaturated end-products and the intermediate high-molecular-weight products confirm that Cdf79930 is an ulvan lyase that cleaves the 1,4-linkage between Rha3S and GlcA or IdoA via the *β*-elimination reaction in an endolytic fashion. The *β*-elimination reaction of lyases proceeds via the abstraction of a proton from the C5 carbon of the uronic acid. This abstracted proton can be in the *syn* (same side) or *anti* (opposite) orientation to the bridging oxygen of the C4 carbon^36^. Previous studies of the chondroitin ABC lyase and heparinase II, which catalyse both *syn* and *anti* proton abstraction because their substrates contain both glucuronic and iduronic acid residues, identified specific amino acid residues that are involved in either *syn* or *anti* abstraction of protons^30,31^. The specificity of the ulvan lyase can be tuned through modification of specific amino acid residues in the protein.

In the present study, we show the presence of an unsaturated uronic acid at the O2 of the terminal rhamnose as a branch. This indicates the ability of the Cdf79930 protein to cleave the structures attached to the branched uronic acid residues. Lahaye *et al*. also observed branching in ulvan at the O2 position^27^. However, details as to the frequency and lengths of the branches are currently not known. Further studies on the branching of ulvan would help to explain its low viscosity and other physicochemical properties.

In contrast to the previously reported ulvan lyases, Cdf79930 from *F*. *agariphila* degraded the glycosaminoglycans chondroitin sulphate and heparan sulphate, despite differences in their compositions. Chondroitin sulphate is mainly composed of N-acetyl galactosamine with 1,3- or 1,4-linked glucuronic acid, whereas heparan sulphate is composed of glucosamine linked to uronic acid. The catalytic actions of this novel ulvan lyase on these glycosaminoglycans indicates less stringency in the specificity of the sugar residue that is linked to the uronic acid.

## Conclusions

Cdf79930, which is a member of the PUL for ulvan, is an evolutionarily distinct endolytic ulvan lyase with broad-spectrum activity against glycosaminoglycans. In contrast to the already known ulvan lyases, this novel enzyme shows a distinct folding pattern, with an (α/α)_6_ incomplete toroid. Therefore, this Cdf79930 lyase is a founding member of a new family of ulvan lyases. Further structure-function studies of this novel ulvan lyase are in progress.

## Experimental Procedures

### Materials

Ulvan from *Ulva amoricana* (winter-heavy) was obtained from Carbosynth (Compton, Berkshire, UK) and used for all the enzyme assays and kinetic studies. Ulvan from *Ulva lactuca* was used for determining the degradation kinetics and identifying the oligosaccharide end-products. It was extracted from *U*. *lactuca* that was collected on the island of Saltö, close to Strömstad, Sweden, using a hot water extraction method with ethanol wash and enzymatic purification, combining published protocols (manuscript in preparation)^16,37–39^.

### Homology modelling and domain architecture

The homology modelling of Cdf79930 was performed using the Phyre2 (**P**rotein **H**omology/analog**Y R**ecognition **E**ngine ver. 2.0) web server set for intensive mode, and the RaptorX homology modelling server^40,41^. The Phyre2 homology model was majorly constructed using the structures of heparinase-III (PDB id- 5jmd), α-mannosidase (PDB id-4c1s), α-1,6 mannanase (PDB id-4v1s), a putative glycosylhydrolase (PDB id-4mu9), and heparinase-iii (PDB id-4fnv). The generated models were saved as PDB files. The structures were then refined using the 3Drefine online server^42^. UCSF-Chimera (https://www.cgl.ucsf.edu/chimera/) was used to visualise the three-dimensional structures^43^. The domain analysis was also carried out using the dbCAN tool available on CAZy database^44^.

### Cloning, expression, and purification

A codon-optimized version of the BN863_22180 gene (protein GenBank no. CDF79930), which encodes 15–622 residues of ulvan lyase, was cloned without a signal peptide into a modified pET28a expression vector (i.e., having a TEV protease cleavage site instead of a thrombin cleavage site), so as to place a 6 × histidine tag at the C-terminus. The resulting recombinant plasmid was transformed into *Escherichia coli* BL21 (DE3) for expression. The overnight inoculum of *E*. *coli* BL21 (DE3) harbouring pET28a-Cdf79930 was transferred to 1 litre of LB broth that contained 50 µg/ml kanamycin, and the culture was incubated at 37 °C. After the OD_600_ of the culture reached 0.8, the incubation temperature was reduced to 16 °C, and the expression of the recombinant protein was induced by the addition of IPTG to a final concentration of 0.2 mM. After induction for 16 hours, the cells were harvested by centrifugation at 10,000 × g for 10 min at 4 °C and resuspended in a lysis buffer [20 mM Tris buffer (pH 8), 500 mM NaCl]. The cells were lysed by ultrasonication for 2 min with alternate cycles of 2 seconds on and 5 seconds off at 20% amplitude. The soluble fraction was collected by centrifugation at 12,000 × *g* for 20 min and loaded onto a 5-ml Histrap-Excel column (GE Healthcare Life Sciences, Chicago, IL, USA) attached to the Äkta purifier (GE Healthcare Life Sciences). The unbound protein was washed with lysis buffer that contained 60 mM imidazole. The bound 6 × His-tagged Cdf79930 was eluted with elution buffer [20 mM Tris (pH 8), 500 mM NaCl, 500 mM imidazole]. The collected eluted fractions were concentrated using Centricon concentrators with a molecular weight cutoff membrane of 10 kDa (Millipore Corp., Burlington, MA, USA). Each concentrated sample was loaded onto a HiPrep™ 16/60 Sephacryl S-100 HR size exclusion column (GE Healthcare Life Sciences) that was equilibrated with a buffer that contained 20 mM Tris buffer (pH 8) and 150 mM NaCl. The eluted protein was collected into two fractions. Fraction 1 contained contaminant proteins, whereas lyase activity was observed in Fraction 2. The fraction with ulvan lyase activity was concentrated to 60 mg/ml using a Centricon (Millipore) concentrator with a 10 kDa molecular weight cutoff membrane and analysed on a TGX Stain-Free gel (4–15% acrylamide; Bio-Rad Laboratories, Hercules, CA, USA).

Circular dichroism spectra (far-UV range) for the purified Cdf79930 sample in 10 mM Tris buffer (pH 7, 8 and 9) were recorded at temperature 20 °C, 40 °C and 96 °C on a Chiroscan instrument (Applied Photophysics Ltd., Leatherhead, Surrey, UK) using a quartz cuvette with a path length of 1 mm. The CD signal of the buffer was subtracted from the average of three different spectra of the sample, and the resulting data were analysed for secondary structure using the K2D2 server (http://cbdm-01.zdv.uni-mainz.de/andrade/k2d2//index.html)^45^. Thermal denaturation of the Cdf79930 was carried out in 10 mM Tris buffer (pH 7, 8, and 9) at 222 nm on a Chiroscan instrument equipped with a Peltier thermostat (Applied Photophysics Ltd., Leatherhead, Surrey, UK) using a quartz cuvette with a path length of 1 mm.

### Enzyme assays

The polysaccharide lyase activity was measured using ulvan as substrate. The ferricyanide method described by Fer *et al*. (2012) was used to quantify the enzyme-generated reducing ends^46^.

To test the substrate specificity of Cdf79930, 200 µg of chondroitin sulphate, heparan sulphate, xanthan, pectin and alginate in 20 mM Tris buffer (pH 8.0) were mixed with 20 µg of Cdf79930 and incubated at 37 °C for 60 minutes. Appropriate controls, i.e., a buffer blank, enzyme blank, and substrate blank, were treated similarly. The free reducing ends were measured using the ferricyanide method.

### Separation of oligosaccharides

Five mg of ulvan dissolved in 20 mM Tris buffer (pH 8.0) with 150 mM NaCl were mixed with 50 µg of the recombinant Cdf79930 and incubated at 37 °C for 12 hours. The lysed mixture was boiled for 5 min to stop the reaction. The samples were centrifuged to remove the protein precipitate, and the supernatant was loaded onto HiPrep™ 26/60 Sephacryl® S-100 HR and Biogel-P2 (100 cm × 1.5 cm) columns connected in series to the Äkta Explorer instrument (GE Healthcare Life Sciences). Separation was carried out using 50 mM ammonium carbonate buffer (pH 8.0) as the eluent, and the separation was monitored with a UV detector operating at 232 nm. The fractions that corresponded to each peak were pooled, freeze-dried, and stored at 4 °C until further analyses.

### NMR analysis

The separated end-products were dissolved in D_2_O (99.97% of atom D). The structures of the separated end-products were determined by ^1^H-NMR and ^13^C-NMR. The ^1^H-NMR and ^13^C-NMR spectra were acquired on a Bruker Avance III HD NMR spectrometer equipped with a TXO cryogenic probe, operating at 800 MHz 1 H frequency, using the solvent residual signal as the chemical shift reference.

### Mass spectrometry

Before the analysis, separated ulvan oligosaccharide samples were reduced by treatment with 0.5 M NaBH_4_ and 20 mM NaOH at 50 °C overnight. The samples were then desalted using a cation exchange resin (AG® 50W-X8 Cation Exchange Resin: Bio-Rad) packed onto a ZipTip C18 tip (34). After drying in a SpeedVac, methanol was added to remove the residual borate by evaporation. The resultant oligosaccharides were dissolved in Milli-Q water at the same volume as the starting material (*ca*. 50 μl) and then analysed using LC-MS/MS in negative-ion mode. The oligosaccharides were separated on a column (10 cm × 250 µm) packed in-house with 5-µm porous graphite particles (Hypercarb; Thermo-Hypersil, Runcorn, Cheshire, UK). The oligosaccharides were injected into the column and eluted with an acetonitrile gradient (Buffer A, 10 mM ammonium bicarbonate; Buffer B, 10 mM ammonium bicarbonate in 80% acetonitrile). The gradient (0–45% Buffer B) was eluted for 46 minutes, followed by a wash step with 100% Buffer B, and equilibrated with Buffer A in the following 24 minutes. A 40 cm × 50 µm i.d. fused silica capillary was used as the transfer line to the ion source. The samples were analysed in negative-ion mode on an LTQ linear ion trap mass spectrometer (Thermo Electron, San José, CA, USA), with an IonMax standard ESI source equipped with a stainless steel needle kept at −3.5 kV. Compressed air was used as the nebuliser gas. The heated capillary was maintained at 270 °C, and the capillary voltage was −50 kV. A full scan (*m/z* 380–2000, two microscans, maximum 100 ms, target value of 30,000) was performed, followed by data-dependent MS^2^ scans (two microscans, maximum 100 ms, target value of 10,000) with normalized collision energy of 35%, isolation window of 2.5 units, activation q 0.25, and activation time of 30 ms). The threshold for MS^2^ was set to 300 counts. Data acquisition and processing were conducted with the Xcalibur ver. 2.0.7 software.

## Electronic supplementary material

Supplementary Information

## Data Availability

The datasets generated during and/or analysed during the current study are available from the corresponding author on reasonable request.
