# Supplementary material for: A novel ulvan lyase family with broad-spectrum activity from the ulvan utilisation loci of Formosa agariphila KMM 3901
Source: Sci Rep. 2018 Oct 2;8:14713. doi: 10.1038/s41598-018-32922-0 (PMC6168547; doi:10.1038/s41598-018-32922-0)
Supplement: Supplementary file 1 — Supplementary Information [file 41598_2018_32922_MOESM1_ESM.pdf]

Supplementary information

A novel ulvan lyase family with broad-spectrum activity from the ulvan utilisation loci of *Formosa agariphila* KMM 3901

Venkat Rao Konasani[1], Chunsheng Jin[2], Niclas G. Karlsson[2], Eva Albers[1]

[1]Industrial Biotechnology Division, Department of Biology and Biological Engineering, Chalmers University of Technology, Gothenburg, Sweden

[2] Department of Medical Biochemistry and Cell Biology, Institute of Biomedicine, University of Gothenburg, Gothenburg, Sweden

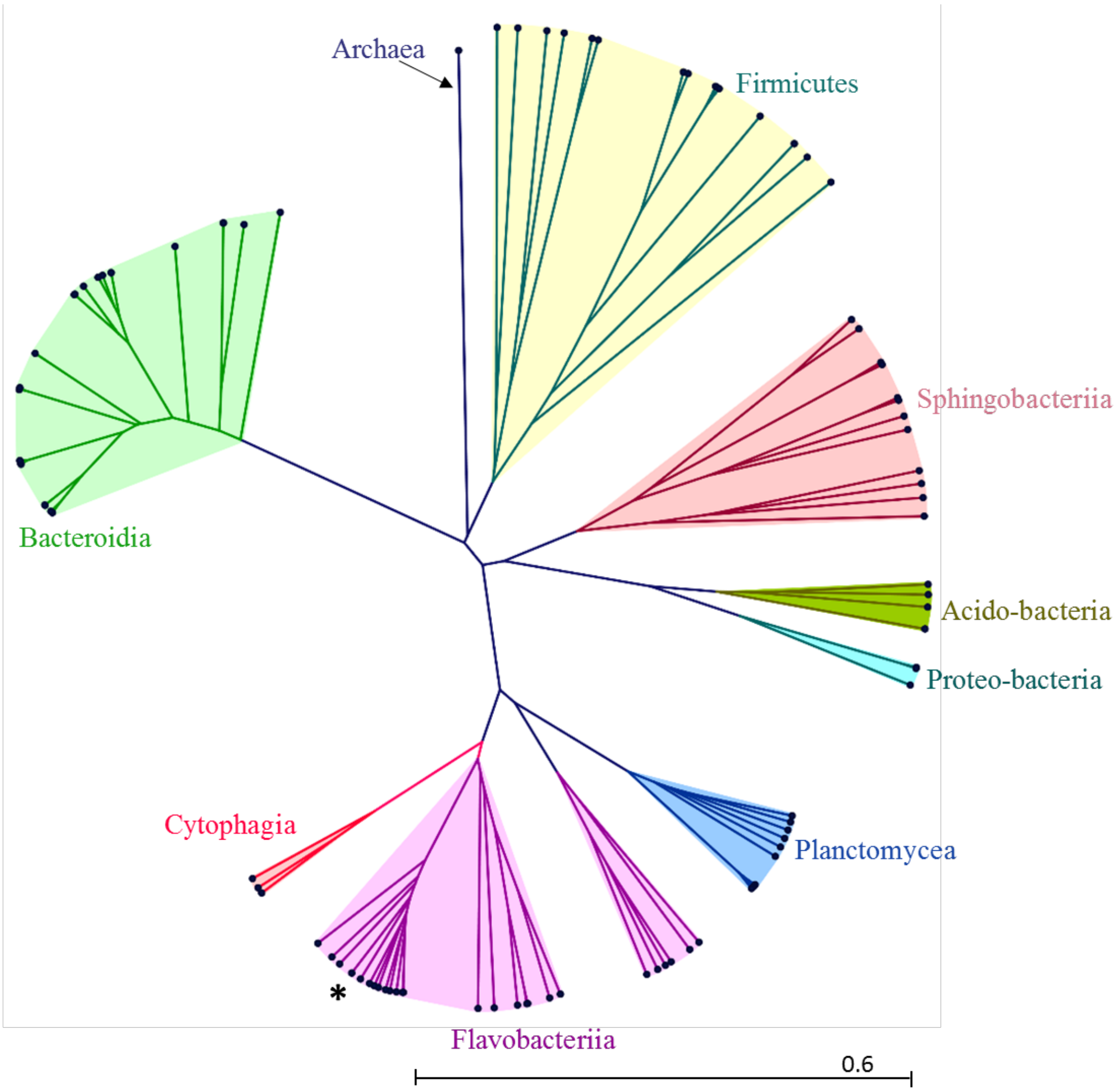

Figure S1. Phylogenetic analysis of homologs of Cdf79930. The homologs were identified using PSI-BLAST, and 143 homologs with >30% identity to Cdf79930 were used in the phylogenetic analysis. The phylogenetic tree was constructed using UPGMA based on the Kimura 2p distance matrix using the CLC Sequence Viewer ver. 8.0 software. Cdf79930 is indicated by the asterisk (*).

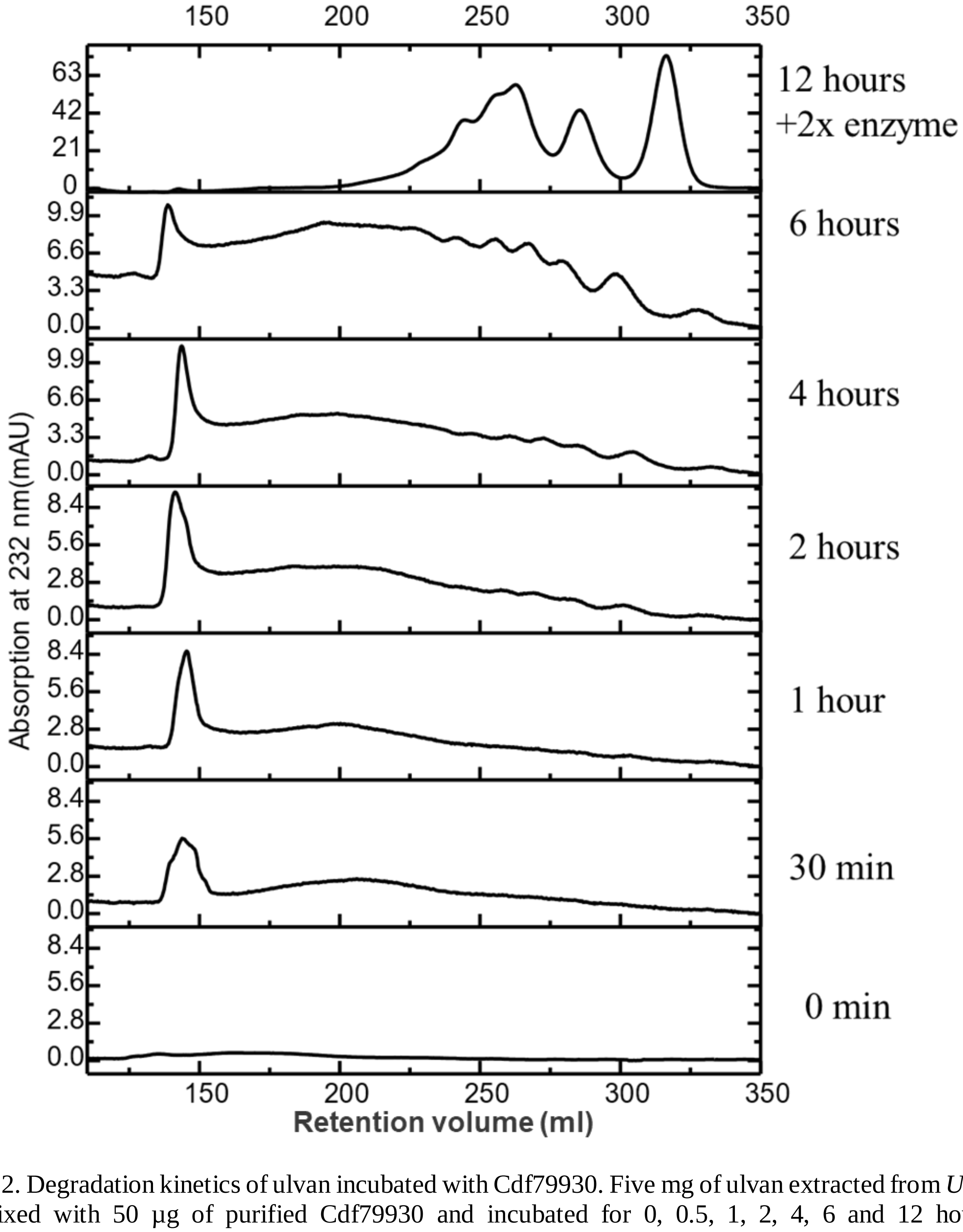

Figure S2. Degradation kinetics of ulvan incubated with Cdf79930. Five mg of ulvan extracted from *U. lactuca* were mixed with 50 µg of purified Cdf79930 and incubated for 0, 0.5, 1, 2, 4, 6 and 12 hours. The oligosaccharide end-products were separated by passage through a size exclusion column.

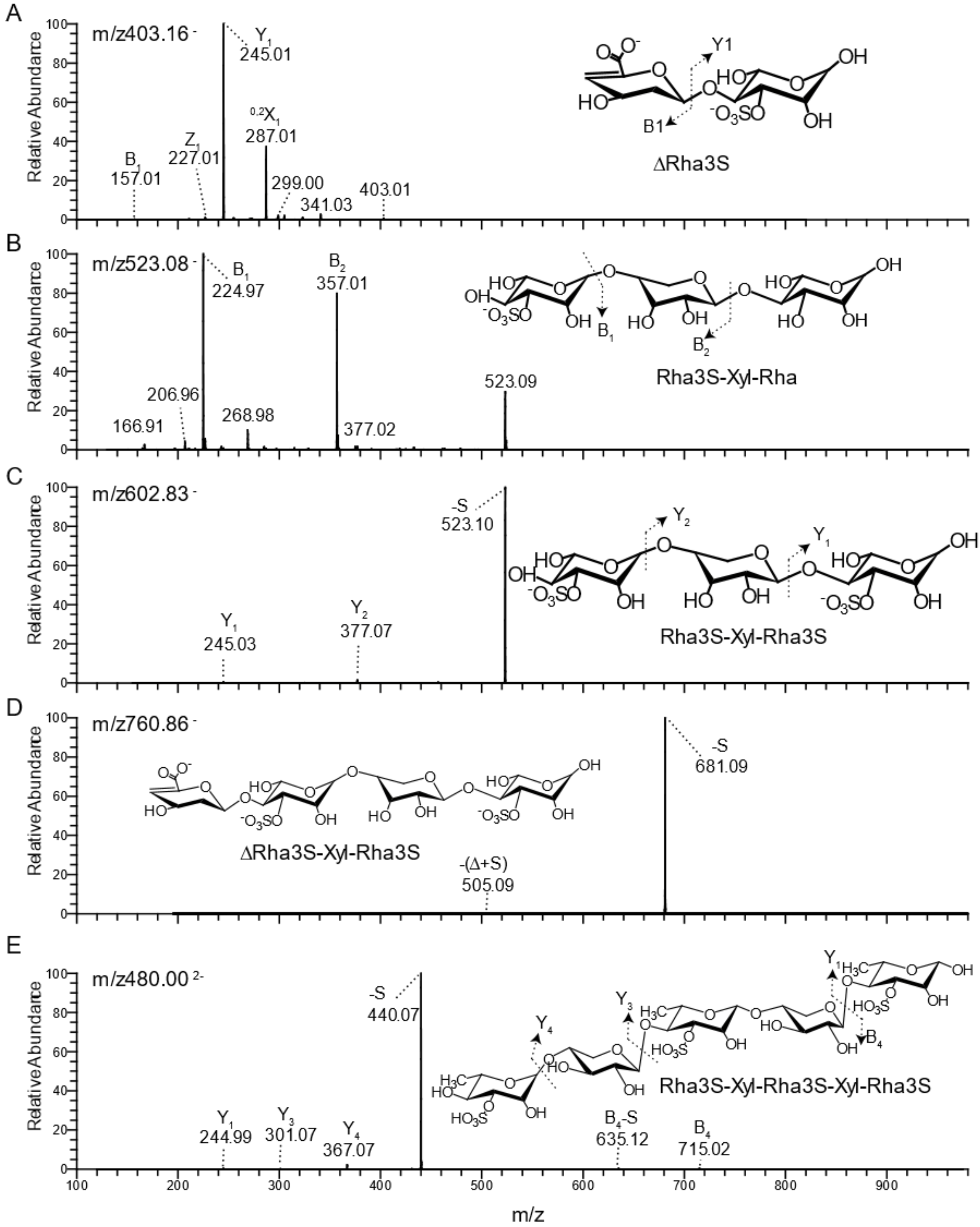

Figure S3. LC-MS/MS analyses of the end-products of Cdf79930-treated ulvan, separated using size exclusion chromatography. Fragmentation analyses reveal: unsaturated disaccharide ΔRha3S (A); trisaccharide Rha3S-Xyl-Rha (B); Rha3S-Xyl-Rha3S (C); tetrasaccharide ΔRha3S-Xyl-Rha3S (D); and Rha3S-Xyl-Rha3S- Xyl-Rha3S (E).

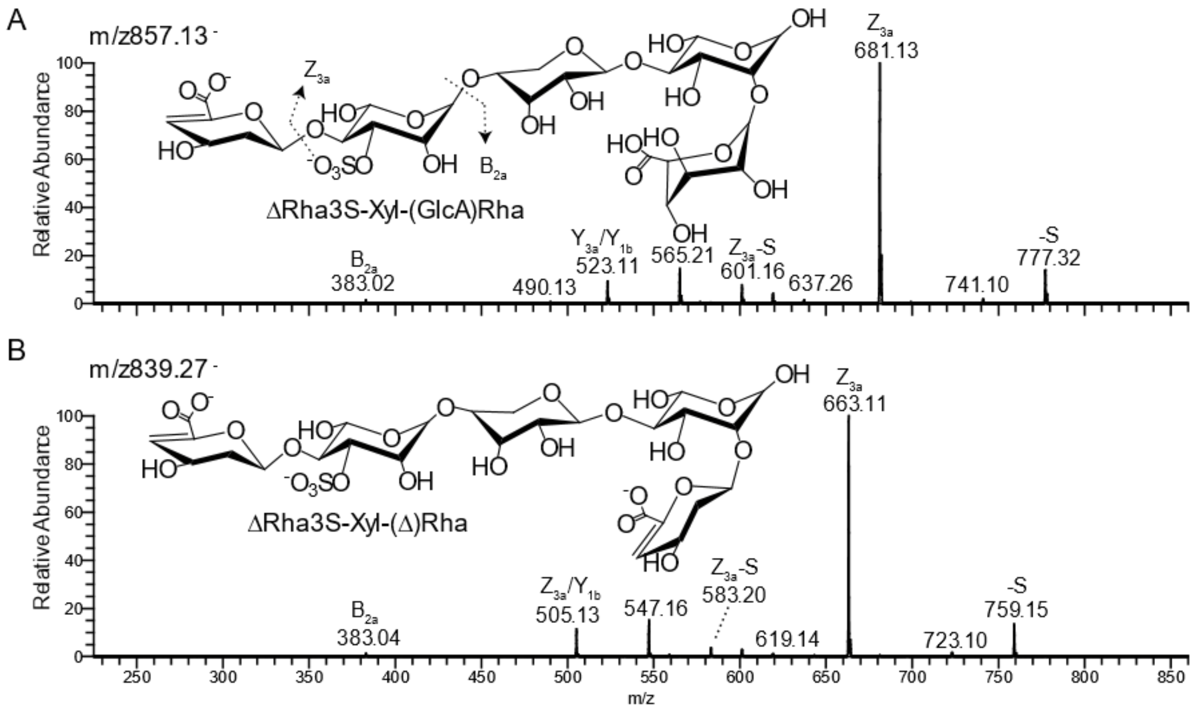

Figure S4. LC-MS/MS analysis of a pentasaccharide from the peak eluted between 258 and 274 ml on size exclusion chromatography. Fragmented mass analysis indicates that the pentasaccharide contains a branched hexuronic acid (A), and that this hexuronic acid is often unsaturated (B) due to the actions of the ulvan lyase on the branches.

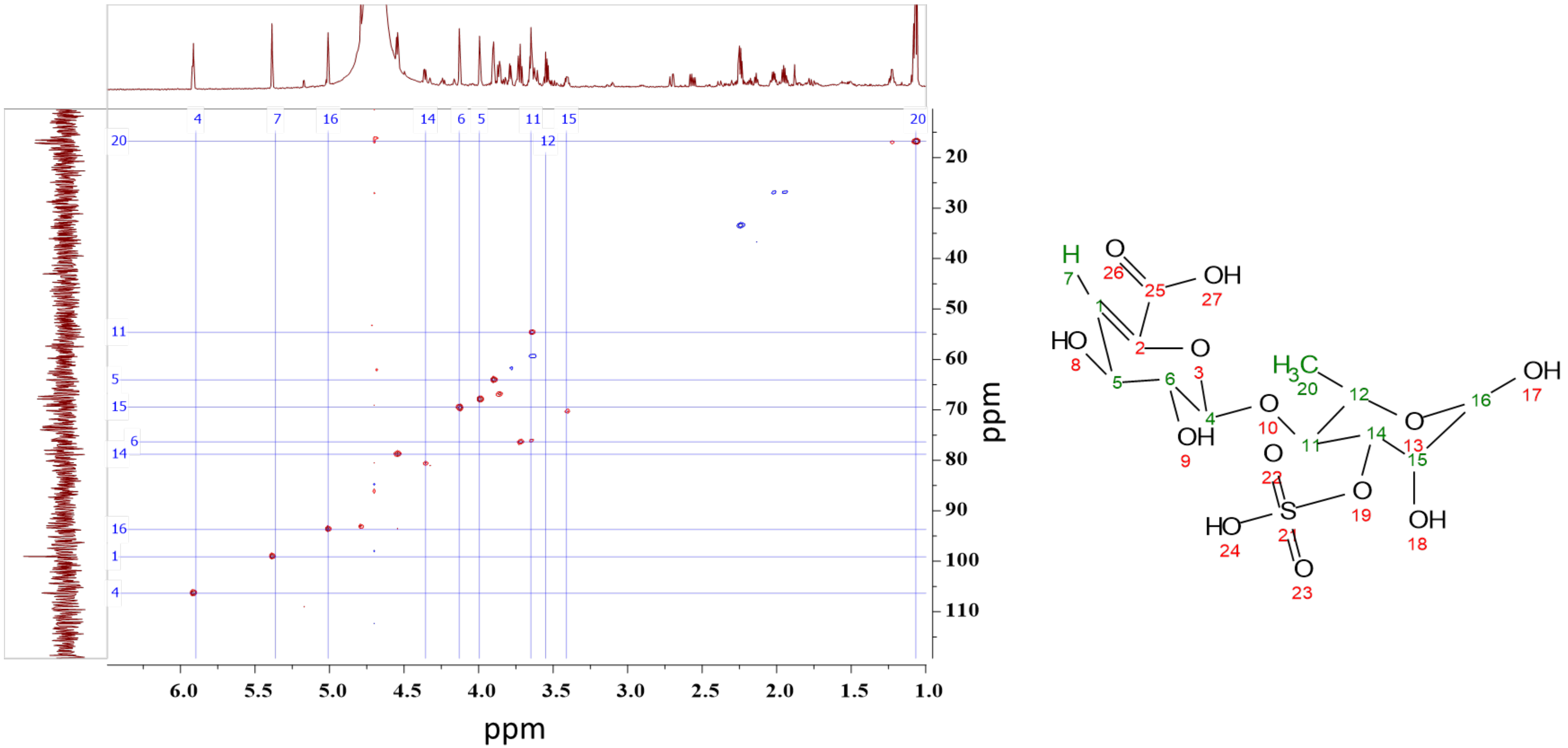

Figure S5. 2D ^{1}H- and ^{13}C-NMR chemical shift correlation spectroscopy (HSQC) contour plot obtained for peak 3 collected during size exclusion chromatography of ulvan treated with Cdf79930. The numbers indicate the assignments for the shifts, as labelled on the structure on the right-hand side.

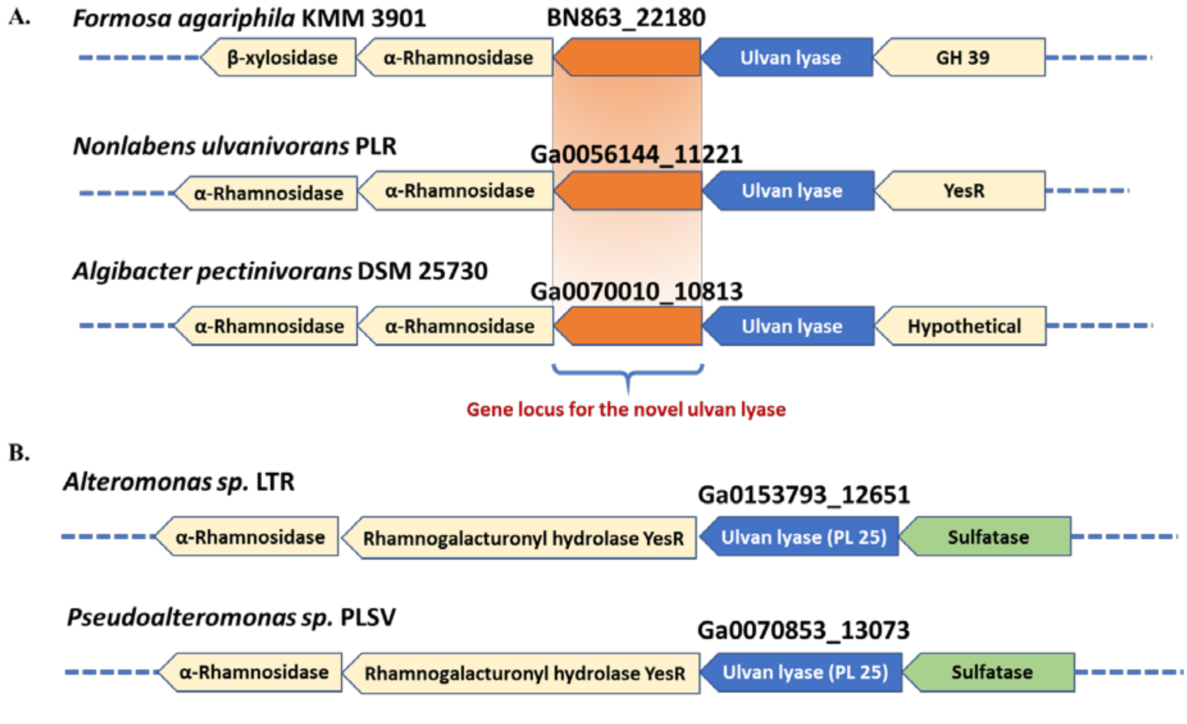

Figure S6. Cdf79930 and the PUL for ulvan. In general, the PUL for ulvan can be classified into two types: (i) *Formosa*, *Nonlabens* and *Algibacter* species have similar PUL and carry homologs of Cdf79930 (**A**); (ii) the PUL of Alteromonadales, which differ from those of *Formosa* and they lack the Cdf79930 homologs (**B**).

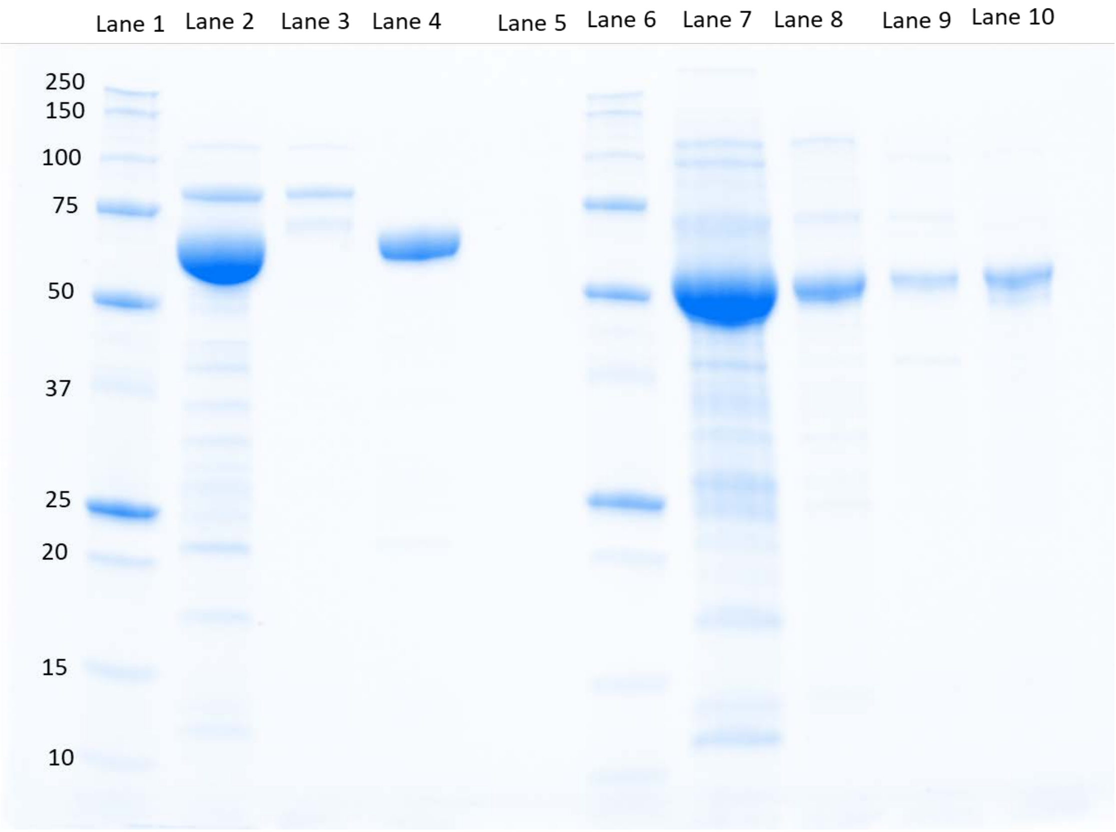

Figure S7. Purification of the Cdf79930. Purification of Cdf79930 by size exclusion chromatography and SDS-PAGE analysis of the purified fractions: lane 1, molecular weight standard; lane 2, Cdf79930 fraction after immobilised metal affinity chromatography; lane 3, fraction 1 after size exclusion chromatography; and lane 4, fraction 2 after size exclusion chromatography. (The remaining lanes 5-10 are of a different protein which is not a part of this manuscript). The gel was not modified except the colour. The colour of the bands was modified from grayscale to Coomassie blue on Bio-Rad ImageLab software.
